# Case report: Intraoperative reversal of flow through an atrial septal defect presenting as hypoxemia during off-pump coronary artery bypass grafting

**DOI:** 10.3389/fcvm.2023.1085755

**Published:** 2023-07-31

**Authors:** Yun Zhao, Chaorui Mi, Juan Xie, Hui Li

**Affiliations:** ^1^ Department of Anesthesiology, The Second Xiangya Hospital, Central South University, Changsha, China; ^2^ Department of Academic Affairs, The Second Xiangya Hospital, Central South University, Changsha, China

**Keywords:** off-pump coronary artery bypass grafting, atrial septal defect, end-tidal carbon dioxide partial pressure, reversal of shunt flow, hypoxemia

## Abstract

The co-existence of atrial septal defect and coronary artery disease in a patient is rare in clinical practice. In the combined surgery of off-pump coronary artery bypass grafting and atrial septal defect closure, the unusual cardiac positions may affect the direction of blood shunting between the atriums, leading to more complex hemodynamic changes. Here, we report a case of a 67-year-old female who underwent refractory hypoxemia related to heart position in such a combined operation.

## 1. Introduction

Coronary artery disease (CAD) is the major cause of mortality and morbidity worldwide (1). In adults, atrial septal defect (ASD) is the most prevalent congenital heart defect (2). In clinical practice, it may occasionally be encountered that these two common diseases are present together in one patient. Previous studies have shown that off-pump coronary artery bypass graft (OPCABG) with intraoperative device closure of ASD is a viable option for some select patients (3). However, in such combined surgery, the unusual cardiac positions of OPCABG may affect the shunt direction of ASD, leading to more complex hemodynamic changes, which have not been reported so far. In this report, we provide the first description of refractory hypoxemia caused by shunt reversal in a patient with ASD undergoing off-pump cardiac surgery.

## 2. Case description

A 67-year-old woman with a history of hypertension and type 2 diabetes mellitus presented to us with symptoms of unstable angina and exertional dyspnea (NYHA class III). Cardiac catheterization revealed triple-vessel disease, including a 60% stenosis of the left main artery, an 85%–90% stenosis of the left anterior descending artery, an 80% stenosis of the diagonal artery, a 90% stenosis of the circumflex artery, and a 60%–90% stenosis of the right coronary artery. Echocardiography was performed, which showed an ASD measuring about 13 mm and unidirectional blood shunting from the left to the right atrium. Due to volume overload, the right ventricle and right atrium dilated, and the mean pulmonary arterial pressure (MPAP) was 28 mmHg. In addition, there was moderate mitral regurgitation as well as mild tricuspid regurgitation. The ejection fraction (EF) was 62%. All other values were within acceptable limits. Considering the comorbidities associated with cardiopulmonary bypass (CPB) and the fact that the ASD was deemed suitable for closure with a percutaneous device, the patient was scheduled for OPCABG and ASD device closure.

**Figure 1:**
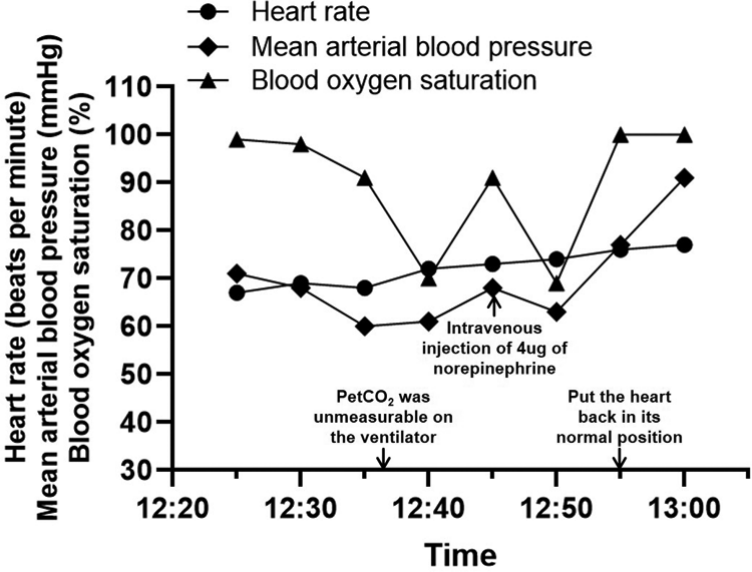
Anesthesia record.

Anesthesia was routinely induced with midazolam, sufentanil, and etomidate and maintained with propofol, remifentanil, and cis-atracurium. Then, we performed a right internal jugular vein puncture, and the patient's baseline central venous pressure (CVP) was measured to be about 10 cmH_2_O. The plan is to graft the left internal mammary artery to the left anterior descending artery and anastomose the saphenous venous to the diagonal, circumflex, and posterior descending branches with the heart beating. The heart was displaced and stabilized during the procedure to expose the target vessel. When the heart was lifted for posterior descending branch distal anastomosis, CVP rose to 20 cmH_2_O, systolic blood pressure (SBP) fell to 79 mmHg, and the heart rate slightly increased. At the same time, the blood oxygen saturation level (SpO_2_) slowly declined from 99% to 91%. We immediately increased the fraction of inspiratory oxygen (FiO_2_) from 60% to 100%, and no problems with the airway, ventilation, or breathing sounds of the patient were reported. However, the SpO_2_ was decreasing even faster, reaching a minimum of 69%. Meanwhile, the end-tidal carbon dioxide partial pressure (PetCO_2_) was unmeasurable by the ventilator. To confirm the SpO_2_ and PetCO_2_, an arterial blood gas analysis was performed, which showed a PaO_2_ level of 40 mmHg and a PaCO_2_ level of 26 mmHg. During this process, a single intravenous injection of 4 µg of norepinephrine could increase BP to above 90 mmHg, and SpO_2_ could also be improved but soon dropped again. In consideration of the altered cardiac anatomy and the elevated right atrial pressure, we assumed there was a shunt reversal with flow from the right atrium into the left and informed the surgical team at once. When the position of the heart was restored to normal, SpO_2_ rapidly went back to 100%, and BP also came back to normal (Figure 1). After coronary revascularization, the ASD was closed with a 16 mm septal occluder under the guidance of transesophageal echocardiography (TEE) in the operating room. The hemodynamics were stable without significant fluctuations during this period. The patient was then taken to the cardiac intensive care unit after the chest was closed. She was successfully extubated on the first day after surgery. According to the postoperative transthoracic echocardiography, the device was in a stable position with no residual shunt. The recovery was uneventful, and the patient was discharged home 7 days later.

## 3. Discussion

ASD is a common non-cyanotic congenital heart defect with a protracted period of asymptomatic development, which accounts for 30% to 40% of heart defects identified in patients over the age of 40 years. Moreover, CAD is the most prevalent acquired heart disease. However, the co-existence of ASD and CAD in a patient is rare in clinical practice. Traditionally, CABG and ASD closure are performed using CPB. With the help of CPB machines, cardiac arrest could provide a clean surgical field of view, allowing the surgeon to perform fine coronary anastomoses. On the other hand, CPB can cause some complications, and ASD trans-atrial closure is more damaging to the heart. Recent studies have demonstrated that device closure of ASD during OPCABG could be a safe and effective alternative technique for some selected patients (4). It can prevent CPB-related injury. For elderly patients, the damage is smaller, the recovery is faster, and the prognosis is better. However, a beating heart can make surgery more difficult and reduce the fineness of surgical operations. In our hospital, OPCABG is routinely preferred because it can avoid CPB-related complications and reduce reperfusion injury. It has also been suggested that while performing the combined procedures, it may be preferable to treat CAD first and then close the ASD because coronary revascularization is expected to enhance ventricular contractility (5) and may affect the occluder position. However, some special hemodynamic changes may occur due to the unusual cardiac positions and the presence of ASD during OPCABG, which have not been reported yet.

In our case, to access the posterior descending branch, the heart had to be elevated and rotated during the procedure. We believed that this unusual position caused a shunt reversal in ASD. On the one hand, blood shunted from right to left through the ASD entered the systemic circulation without being oxygenated by the lungs. On the other hand, the reduced blood flow into the pulmonary circulation led to a reduction in the amount of blood oxygenated through the lungs and an imbalance in V/Q. Both of these reasons led to a decrease in the proportion of oxygenated hemoglobin in the body, resulting in a fall in systemic desaturation. We also observed a peculiar phenomenon—the PetCO_2_ on the ventilator was undetectable, while the PaCO_2_ level in arterial blood gas remained at 26 mmHg. Alveolar ventilation, peripheral CO_2_ production, and pulmonary blood flow all affect PetCO_2_ levels. Since the first two factors are constant during general anesthesia, PetCO_2_ is primarily influenced by pulmonary blood flow. Saleh and Pullan noted that as the heart was elevated to expose the target vessels in OPCABG, PetCO_2_ almost instantly dropped to the low 20 mmHg range and then stabilized or trended back to pre-displacement levels (6). It is consistent with our usual experience. However in this case, PetCO_2_ fell to such a low level that even the ventilator could no longer measure it. It is likely that ASD was also involved in this process. The increased right atrial pressure may have provoked a shunt reversal from the right atrium to the left, leading to a further decrease in pulmonary blood flow and increasing the chance of hemoglobin desaturation.

In addition, previous studies have demonstrated that the Pa-ET CO_2_ can be increased both in cyanotic and non-cyanotic congenital heart diseases. Among them, the cyanotic patients have the highest Pa-ET CO_2_ with a mean of approximately 15 mmHg and a maximum of approximately 20 mmHg (7). In this case, although the patient's preoperative echocardiography showed a unidirectional left-to-right shunt through the ASD, the Pa-ET CO_2_ transiently reached over 20 mmHg during the operation, which meant that a similar shunt direction to cyanotic heart disease may have occurred. This was further evidence that a right-to-left shunt may have existed at that time.

Intraoperatively, the patient had a combination of hypotension in addition to hypoxemia. We believe that the reasons are as follows. During anastomosis of the posterior descending branch, the heart was elevated to a nearly vertical position, and part of the right ventricular free wall was pressed against the ventricular septum. The right ventricular wall was prone to deformation due to its thinness. In early diastole, the left ventricular septum moved toward the right ventricular free wall, further reducing the right ventricular size with a consequent reduction in diastolic return blood volume. In addition, due to the use of cardiac fixators, the systolic and diastolic function of the heart was limited, resulting in a decrease in cardiac output, a decrease in mean arterial pressure, and a compensatory increase in heart rate. For these reasons, hypotension is prone to occur during posterior descending branch anastomosis performed in OPCABG. In this case, because the patient also had an ASD, blood flow not passing through the lung bed may enter the left heart through the ASD during excessive cardiac torsion. However, due to the limitation of the diastolic and systolic function of the heart and the reduction of both return blood volume and cardiac output, systemic hypotension may still occur. Our treatment was to use vasoactive drugs and change the patient to a head-down position to maintain hemodynamic stability.

From this case, we have gained some experience in performing such combined procedures. Before the operation, the patient should be strictly screened for indications and contraindications, and the risk of possible complications should be carefully assessed. For patients with CAD combined with ASD, the advantages and disadvantages of cardiac arrest surgery should be fully considered according to the specific conditions, recognizing the unique benefits of CPB for these patients. If OPCABG and ASD device closure is performed, communication with the surgeon throughout the procedure is required to avoid excessive movement of the heart. When the shunt reversal occurs, intraoperative TEE can provide a fundamental diagnostic tool to detect interatrial shunts (8), which is useful in quickly diagnosing the problem. Furthermore, for this case, we focused on the change in PetCO_2_. Previous work has shown that when there was a decreased cardiac output after repositioning the heart during OPCABG, the change in PetCO_2_ preceded electrical instability and hemodynamics. This method has the benefits of simplicity, general availability, and a quick response time to cardiac output changes (6). If there is a sudden and persistent arterial desaturation during coronary revascularization and TEE or PetCO_2_ indicates the shunt flow going from right to left through ASD, the surgical team must be notified at once and put the heart back in its natural position. Initiating CPB may be considered if desaturation remains severe even after repositioning the heart.

In conclusion, we have described a case of refractory hypoxemia in an OPCABG patient with ASD. For the first time, we report atrial blood flow reversal related to heart position in such combined surgery. When ventilatory causes can be ruled out, the sudden and persistent arterial desaturation associated with cardiac mobilization should be taken as an indication of a possible right-to-left shunt via ASD. The use of intraoperative TEE can be very helpful in confirming the presence of a reverse shunt. In addition, special attention should be paid to the changes in PetCO_2_, which can provide a significant reference for rapid diagnosis.

## Data Availability

The original contributions presented in the study are included in the article, further inquiries can be directed to the corresponding author.
